# Directional topography gradients drive optimum alignment and differentiation of human myoblasts

**DOI:** 10.1002/term.2976

**Published:** 2019-11-10

**Authors:** Ana Maria Almonacid Suarez, Qihui Zhou, Patrick van Rijn, Martin C. Harmsen

**Affiliations:** ^1^ Department of Pathology and Medical Biology, University Medical Center Groningen University of Groningen Groningen The Netherlands; ^2^ Department of Biomedical Engineering, W.J. Kolff Institute for Biomedical Engineering and Materials Science, University Medical Center Groningen University of Groningen Groningen The Netherlands; ^3^ Zernike Institute for Advanced Materials University of Groningen Groningen The Netherlands

**Keywords:** myoblasts, myotubes, polydimethylsiloxane (PDMS), tissue engineering, topography gradient

## Abstract

Tissue engineering of skeletal muscle aims to replicate the parallel alignment of myotubes on the native tissue. Directional topography gradients allow the study of the influence of topography on cellular orientation, proliferation, and differentiation, resulting in yield cues and clues to develop a proper in vitro environment for muscle tissue engineering. In this study, we used a polydimethylsiloxane‐based substrate containing an aligned topography gradient with sinusoidal features ranging from wavelength (λ) = 1,520 nm and amplitude (A) =176 nm to λ = 9,934 nm and A = 2,168 nm. With this topography gradient, we evaluated the effect of topography on human myoblasts distribution, dominant orientation, cell area, nuclei coverage, cell area per number of nuclei, and nuclei area of myotubes. We showed that human myoblasts aligned and differentiated irrespective of the topography section. In addition, aligned human myotubes showed functionality and maturity by contracting spontaneously and nuclei peripheral organization resembling natural myotubes.

## 1 INTRODUCTION

Skeletal muscle is one of the tissues of the body with regenerative capacity. After skeletal muscle injury, the endogenous muscle stem cells, satellite cells, are activated to recover the lost myofibers (Juhas, Ye, & Bursac, 2016). However, large trauma or other causes such as facial palsy demand replacement with (tissue) engineered skeletal tissue. Tissue engineering of skeletal muscle essentially replicates physiological musculogenesis (Shadrin, Khodabukus, & Bursac, 2016) albeit at larger scale. Skeletal muscle has a highly organized architecture that comprises parallel arranged bundles of myofibers of multiple contractile myotubes. Myotubes are syncytia that are derived from fusion of activated myoblasts. Functional, contractile skeletal muscle is innervated by motor neurons and perfused by a vascular network whereas myofibers and muscle is constrained by a fascicle (Gillies & Lieber, 2012; Shadrin et al., 2016). The parallel alignment of muscle (sub)structures, renders it suitable for topography‐guided tissue engineering.

Our previous research (Koning, Werker, van der Schaft, Bank, & Harmsen, 2012; Koning, Werker, van Luyn, & Harmsen, 2011; Koning, Werker, van Luyn, Krenning, & Harmsen, 2012) showed that the biological properties of human myoblasts differ strongly from the “Gold Standard” C2C12 murine myoblast cell line (Koning, Harmsen, Van Luyn, & Werker, 2009). Therefore, research aimed at engineering replacement muscle should focus on primary human myoblasts. We showed that the microRNAs dictate both differentiation and quiescence in satellite cells (Koning, Werker, van der Schaft, et al., 2012; Koning, Werker, van Luyn, et al., 2012), although hypoxia is a strong inducer of their proliferation (Koning et al., 2011). The influence of the substrate was not assessed in our previous studies and it still remains underexposed in current literature.

The muscle cells' natural substrate is the extracellular matrix (ECM) that comprises a biochemical microenvironment consisting of mostly fibrous proteins and negatively charged polysaccharides (Li, Xiao, & Liu, 2017). The ECM also comprises a physical, topographical microenvironment that augments architectural guidance of adhered cells (Watt & Huck, 2013). Material composition, physical and chemical properties, architectural properties as geometry, and topography can be manipulated to resemble natural tissues ECM (Huang et al., 2017; Keung, Healy, Kumar, & Schaffer, 2010; Li et al., 2017).

The manipulation of surface topography facilitates cell alignment and differentiation of myoblasts toward skeletal muscle myotubes (Kim et al., 2013) albeit that material composition and topographical cues have only been studied ad hoc for murine myoblasts (Jana, Levengood, & Zhang, 2016). Nanopatterned substrates with different architectures varying from 50 to 50 μm in height, 800 to 50‐μm ridge, and 800 to 200‐μm width/groove have been used to align C2C12 cells (ridge and width together are called “pitch”; Altomare, Gadegaard, Visai, Tanzi, & Farè, 2010; Bettadapur et al., 2016; Charest, Garcia, & King, 2007; Duffy, Sun, & Feinberg, 2016; Fujie et al., 2015; Grigola, Dyck, Babacan, Joaquin, & Hsia, 2014; Lam, Sim, Zhu, & Takayama, 2006; Murray, Nock, Evans, & Alkaisi, 2016; Ostrovidov et al., 2017; Yamamoto et al., 2008; Yang et al., 2014). In constrast, few studies with human myotubes have been performed (Choi, Lee, Christ, Atala, & Yoo, 2008; Duffy et al., 2016; Nagase et al., 2017; Takahashi, Shimizu, Nakayama, Yamato, & Okano, 2015). It has been shown that in surface protein patterns, human cells align and behave different compared with those of murine origin (Duffy et al., 2016; Sengupta, Gilbert, Johnson, Blau, & Heilshorn, 2012) and showed alignment at 20 μm or higher protein line wavelengths (Duffy et al., 2016).

Cast ECM‐based hydrogels with embedded myoblasts and fixed at their termini were investigated for their propensity to build up pulling tension (Madden, Juhas, Kraus, Truskey, & Bursac, 2015). In these 3D systems, alignment of myotubes occurred, yet these were randomly scattered in the gels without formation of full size muscle fibers. This indicates that the 3D systems are not adequately providing alignment guidence.

Model substrates consisting of linearly aligned topography nanometer to micrometer‐sized gradients in polydimethylsiloxane (PDMS, silicone rubber) are useful to investigate biological features such as adhesion, proliferation, morphology, and differentiation of (stem) cells (Zhou et al., 2017, 2015a). In addition, these 2D systems, more than 3D systems, add to understand the role between the topography and muscle formation (Duffy et al., 2016). An efficient, fast, economic, and reproducible procedure to generate these topographies, that is, “wrinkle” gradients in sheets of cast flat PDMS, is shielded surface oxidation with air plasma (Jana et al., 2016). This technique generates sinusoidal substrates in which the amplitude of the features increases with increasing wavelength “pitch”. The gradient is generated by using an angle mask that provides spatial control over the surface oxidation (Zhou et al., 2015a).

We hypothesized that primary human myoblasts adhere and proliferate in a preferred surface topography, whereas this also promotes fusion, maturation, and alignment of myotubes.

## 2 MATERIALS AND METHODS

### 2.1 Fabrication of the directional topography gradient

PDMS gradients were made following our previously published protocol (Zhou et al., 2016). Briefly, commercially available two component kit Dow Corning, consisting of an elastomer (Sylgard‐184A) and a curing agent (Sylgard 184B), were mixed at ratio of 10: 1 w/w. The mixture was degassed by applying vacuum, and 20 g was poured in a 12 × 12‐cm polystyrene petri dish after which it was cured at 70°C overnight. After curing, PDMS films were cut in pieces of 2.5 × 2 cm. Each piece was placed in a custom‐made stretching device and stretched 30% of its original length. To generate a topography gradient, a triangle‐shaped metal mask of 1.3 cm long and 1.0 cm wide with an angle of 30° was placed on top of the stretched PDMS substrate. Then, the system was placed in a plasma oven (Diener electronic, model Atto, Ebhausen, Germany) for surface oxidation with air plasma at 10 mTorr for 600 s at maximum power. Subsequently, the tension on the PDMS substrate was carefully removed by releasing the stress gradually from the custom‐made stretching device. Upon reduction of tension, wrinkled topography is formed with large wrinkles on the open side of the mask, which progressively become smaller toward the closed side of the mask. Finally, to reach a uniform oxidation state of the surface to ensure a homogenous stiffness, gradients were placed again under air plasma at 130 mTorr pressure for 600 s at maximum power.

### 2.2 AFM characterization of topography gradient

Atomic force microscopy (AFM) contact‐mode measurements were performed on a Catalyst NanoScope IV instrument (Bruker, Billerica, MA, USA) with NanoScope Analysis (Bruker Billerica, MA, USA) as analysis software. Cantilever “D” from DNP‐10 Bruker's robust Silicon Nitride AFM probe was used. AFM was performed for on duplicates of three independent made PDMS gradient samples. Each sample was analyzed on three different points on each of the sections on gradient surface.

### 2.3 Sterilization of surfaces

A 1.8‐cm^2^ circle‐shaped PDMS pieces containing the 1 × 1‐cm gradients were washed with 70% ethanol in culture plates followed by a second ethanol wash, which was left for 10 min. For removing traces of ethanol, the PDMS gradients were washed with phosphate‐buffered saline (PBS).

### 2.4 Cell culture of myoblasts

Myoblasts used were isolated from our previous studies (Koning, 2012). Briefly, myoblasts were cultured from collagenase‐treated muscle biopsies of the orbicularis oculi muscle (five donors) of human donors (51.7 ± 10.6 years) undergoing reconstructive surgery (Koning, 2012). These myoblasts had a high self‐renewal and cloning capacity. Clones were obtained as previously described (Koning, Werker, van der Schaft, et al., 2012; Koning, Werker, van Luyn, et al., 2012). Briefly, when isolated cells were at Passage 8, cells were sorted by using MoFlow FACs on a 96‐well plate. Clones expressing cell markers Pax7, MyoD, and Myogenin were selected for experiments. For the current studies, clone V49 was used between Passage 5 and 8. V49 cells were maintained on gelatin‐coated plates in high glucose Dulbecco's Modified Eagle's Medium (Sigma‐Aldrich/Merck KGaA, Darmstadt, Germany), L‐Glut, 20% fetal bovine serum (Life Technologies Gibco/Merck KGaA, Darmstadt, Germany), and 1% penicillin/streptomycin (Sigma‐Aldrich/Merck KGaA, Darmstadt, Germany), that is, proliferation medium (PM). Cells were passaged at a 1:3 ratio after detachment with Accutase (Sigma‐Aldrich/Merck KGaA, Darmstadt, Germany). For experiments, V49 myoblasts were seeded on tissue culture plastic or PDMS substrates (see below) at 5,000 per square centimeter in PM. Upon reaching confluence, medium was changed to differentiation medium (DM), composed of Dulbecco's Modified Eagle's Medium, 2% fetal bovine serum, 1% penicillin/streptomycin, 1% insulin‐transferrin‐selenium (Gibco by Life Technologies/Merck KGaA, Darmstadt, Germany), and 1% dexamethason (Sigma‐Aldrich/Merck KGaA, Darmstadt, Germany).

For examination of cells during the experiments, an inverted microscope Leica DM IL LED equipped with DFC 425C CCD camera and the software LAS V4.5 (Leica Microsystems CMS GmbH, Wetzlar, Germany) was used.

### 2.5 Experimental design

**Figure 1:**
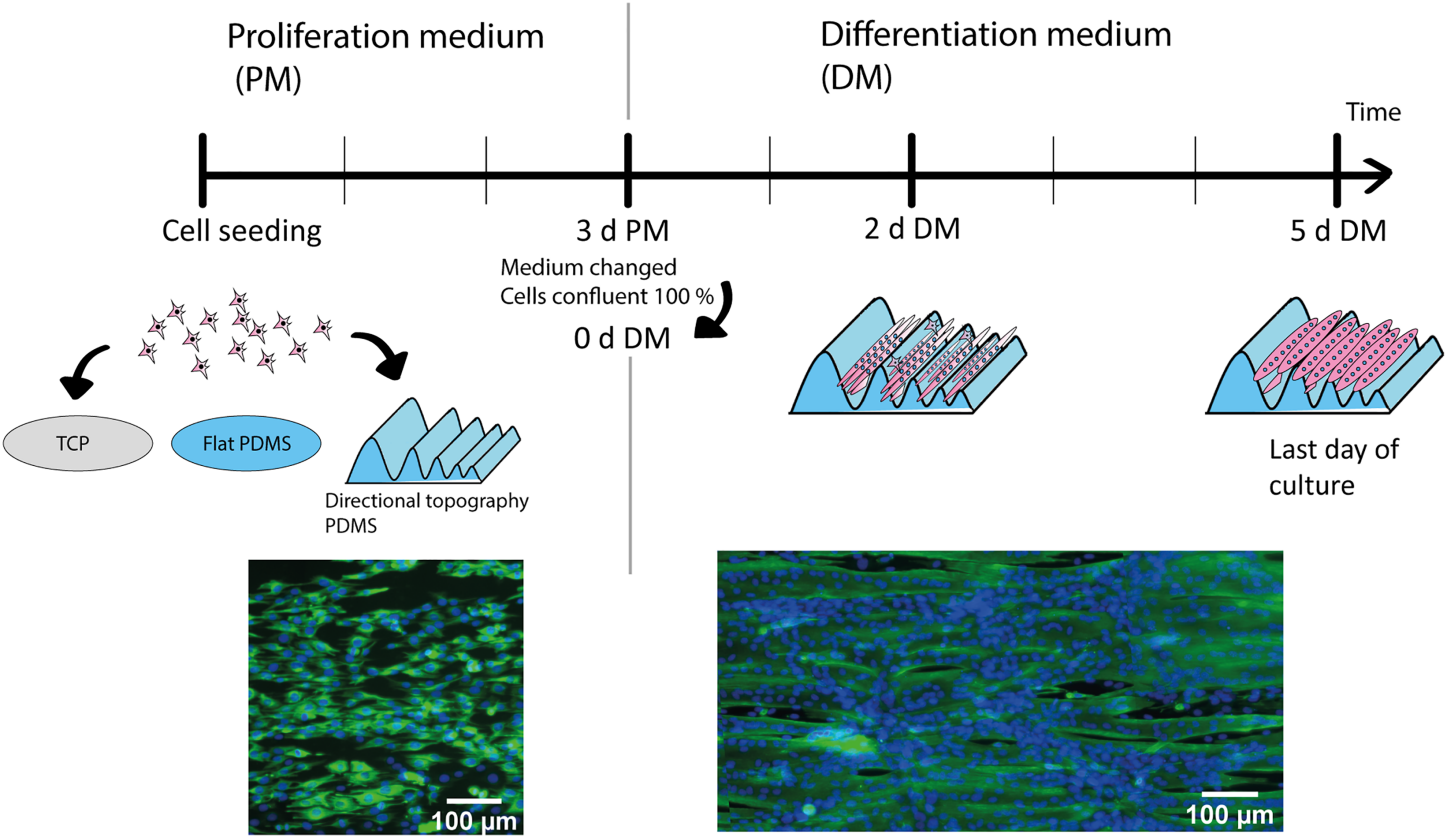
Experimental design. Myoblasts were seeded and left for 3 days in culture in the different materials. Differentiation medium was added once cells were 100% confluence in the tissue culture polystyrene. Myotubes were differentiated for 5 days. Desmin (green) and DAPI (blue). [Colour figure can be viewed at http://wileyonlinelibrary.com]

Three independent experiments with three technical replicates per experiment were performed. Samples consisted of tissue culture polystyrene and flat PDMS, as controls, and topography gradients in PDMS. All cell cultures were made in 24‐well plates with a culture area of 2 cm^2^. Round PDMS samples (2 cm^2^) were cut and sterilized with ethanol, 70% ethanol. Cells adhesion, proliferation, and differentiation were evaluated prior to initiating differentiation (t 0) and at 2 and 5 days of applying differentiation conditions (Figure 1).

### 2.6 Immunofluorescence staining

Immunofluorescence staining was done after 3 days in PM and 2 and 5 days in DM. After three PBS washes, cells were fixed in 2% paraformaldehyde in PBS at room temperature for 20 min, washed two times with PBS, and stored at 4°C. For staining, cells were permeabilized with 0.5 % Triton X‐100 (Sigma‐Aldrich/Merck KGaA, Darmstadt, Germany) in PBS at room temperature for 10 min followed by PBS wash. Then, nonspecific binding sites were blocked with 10% donkey serum in PBS for 30 min. Cells were incubated with rabbit anti‐human desmin (1:100) antibodies (NB120‐15200, Novus Biologicals, Abingdon, England) or mouse anti‐human Myosin heavy chain (MCH) (1:20; MF 20 was deposited to the Developmental Studies Hybridoma Bank by Fischman, D.A. [Developmental Studies Hybridoma Bank Hybridoma Product MF 20] in PBS with 2% bovine serum at room temperature for 60 min). One sample was left without primary antibody to be used as staining control for nonspecific binding of the secondary antibody to the specimen. Next, three washes with 0.05 % Tween‐20 in PBS were performed. Finally, samples were incubated with donkey anti‐rabbit IgG (H + L) Alexa Fluor® 488 or donkey anti‐mouse Alexa fluor 488 (Life technologies; 1:300), (1‐μg/ml DAPI) in PBS with 5% normal human serum for 30 min. Nonbound antibodies were removed by subsequent washes with 0.05% Tween‐20 in PBS and PBS wash. Samples were stored in PBS 1% penicillin/streptomycin at 4°C.

Immunofluorescence imaging was done by fluorescence microscopy using the TissueFAXS imaging setup with a Zeiss AxioObserver.Z1 microscope and TissueQuest cell analysis software (TissueGnostics, Vienna, Austria). The micrographs obtained by the TissueFAXS analyses were stitched together to yield an image that covered the entire topography gradient.

### 2.7 Image analysis with Image J

Cell analysis across the topography gradient for each sample was done from an image covering 4 mm in width and the entire length of the gradient (10 mm). The length was divided in 10 sections of 1 mm. These sections (1 to 10) each represent a specific range of wavelengths and amplitudes, ranging from nanometer to micrometer sizes. The images were analyzed with Image J to determine the nuclear area (DAPI), myotube diameter, and percentage of area covered by cells (desmin expression) after 3 days in PM and 2 and 5 days in DM.

The average size of the DAPI‐stained nuclei was determined by adjusting the image threshold manually to ensure that nuclei area was being taken properly. Then, image was made binary and “watershed” was applied to distinguish between clustered nuclei. Next, “particle analyzer” was implemented within a size range of 20 to 400 μm^2^ to avoid counting of clusters that could not be removed by watershed.

Myotube diameters were measured manually using the ‘line and freehand’ selection tool of Image J. At least 25 distinct myotubes, chosen randomly, were evaluated per sample for a total of 100 measurements per treatment and experiment. Every section (1–10) of the gradient was analyzed corresponding to images with areas of 1 × 4 mm. Then, the different wavelengths and amplitudes, previously measured with the AFM, were related with the myotubes' diameter, as all spot‐specific features are known on the entire directional topography surface.

Cellular and nuclear distribution among the gradient was measured by the expression (fluorescence) level of respectively desmin and DAPI. This yielded the percentage area covered by these colors, which shows cellular and nuclear spread on the gradients per section in the different time points. Briefly, images were color split, and “auto threshold” (Otsu dark) was chosen to measure the percentage area covered.

Cell area per number of nuclei or fusion ratio was calculated from the measurement of the cell area covered by cells expressing desmin and number of nuclei in the same gradient section. Nuclei area was considered constant, assumption made by resulted measurements. Then, the area of cells expressing desmin was divided against the number of nuclei in that region.

### 2.8 Statistical Analysis

Data were assessed for normality with the Shapiro–Wilk normality test. One‐way analysis of variance (ANOVA) was done to check differences within the section of the gradient, and Tukey's multiple comparison test was used to analyze significant difference within topographical features for dominant orientation, cell area and nuclei coverage, and cell area per number of nuclei and nuclei area. Two‐way ANOVA and Tukey's multiple comparison test were used to analyze the interaction between the different time points (cellular maturity) and the different features from the topography. Row and columns factors were also analyzed. Significance was considered when *p* < .05. Analysis of myotube diameter was done by Kruskal–Wallis and Wilcoxon matched‐pairs signed rank test as data did not pass the normality test. Data analysis was carried out using GraphPad Prism 6 (GraphPad software, La Jolla, CA).

## 3 RESULTS

### 3.1 Alignment of myotubes occurs in all topographical features

**Figure 2:**
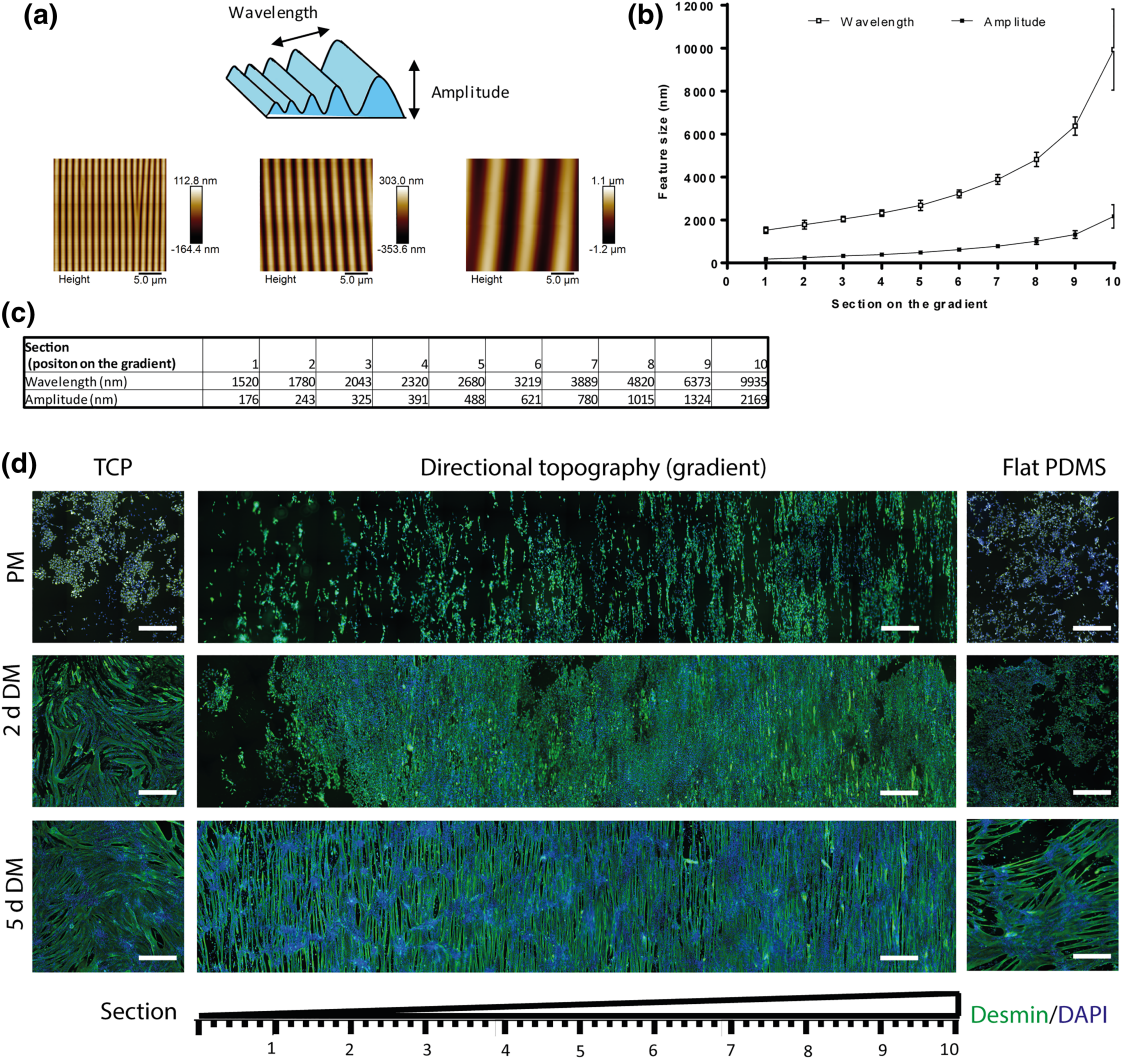
(a) Schematic representation and atomic force microscopy images of linear topographical gradients at Sections 1, 5, and 10. (b) Atomic force microscopy‐generated wavelength—high characterization of the PDMS‐based topography gradients. (c) Description of the different sections on the gradient and its correspondent value of wavelength and amplitude (nanometer). (d) Micrographs of myoblasts cultured in PM for 3 days. Myoblasts were spread and aligned among the gradient although on TCP and flat PDMS, controls (substrates without topography) had no orientation. After 2 days of differentiation (2‐days DM), a mixed cell population of myoblasts and primitive of myotubes occurred. Following 5 days of differentiation (5‐days DM), myotube formation was visible in all directional topography sizes and in the different flat controls. Gradient PDMS substrate with Section 1 to 10. Scale bars represent 500 μm. Green is desmin, and blue is DAPI (nuclei). DM, differentiation medium; PDMS, polydimethylsiloxane; PM, proliferation medium; TCP, tissue culture polystyrene [Colour figure can be viewed at http://wileyonlinelibrary.com]

The plasma treatment of stretched PDMS generated a directional topography gradient with a sinusoidal shape with features altering across the gradient surface with wavelengths (λ i.e., pitch) and amplitude (A i.e., height) from λ = 1,520 nm and A = 176 nm (Section 1) to λ = 9,934 nm and A = 2,168 nm (Section 10; Figure 2a,b,c). Sections 1 to 10, respectively, are the first to the last millimeter of the gradient. Stiffness was constant among all sections of the gradient.

**Figure 3:**
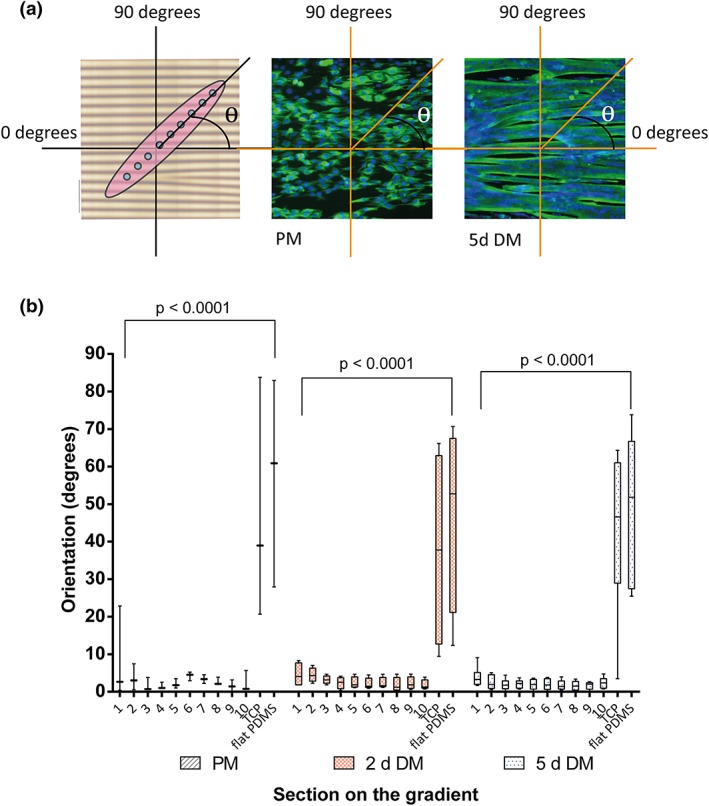
Orientation of cells during proliferation and differentiation. (a) The angle of alignment of the cells and myotubes on the area of the gradient corresponding to the different sections was measured and averaged. An angle closer to 0° indicates a higher alignment of cells or myotubes to the linear topography. Angles were measured always between 0° and 90° as depicted in the figure. Angles were always considered positive and less than 90°. Micrographs depict angle measurement of myoblasts alignment after 3 days in PM and myotubes after 5 days in DM. Scale bars represent 150 μm. Cells were visualized by immunofluorescence staining for desmin (green) and nuclei with DAPI (blue). (b) One‐way analysis of variance (PM *p* < .0001, 2 days in DM *p* < .0001, and 5 days in DM *p* < .0001). Data represented by box and whiskers plotting the minimum (smallest value) to the maximum (largest value) values and the line at the median. DM, differentiation medium; PM, proliferation medium [Colour figure can be viewed at http://wileyonlinelibrary.com]

Myoblasts were all aligned on the directional topography after 3 days in culture (Figure 2d, PM; Figure 3a). Proliferation was lower on PDMS surfaces (topography and flat) in comparison with the tissue culture polystyrene (TCP). Once the TCP monolayer was confluent, the medium was changed to differentiation medium. Then, the myoblasts started to fuse and formed myotubes. After 2 days of differentiation, a mixed population of myoblasts and myotubes were found on all surfaces. It was specially visible in aligned myoblasts and myotubes (Figure S1). The cells residing on the directional topography were aligned and were following the direction of the topography. However, cellular behavior varied between the different substrates. The flat PDMS presented less myotube formation than on TCP. Once cells were differentiated for 5 days (Figure 2d, 5‐days DM), myotubes were observed on all substrates. Similarly, TCP and PDMS had disorganized myotubes. TCP presented stable cell attachment unlike flat PDMS that had less cells attached and was prone to detachment of cells. On the other hand, myotubes on the directional topography were following the linear pattern irrespective of the section on the gradient and presented a more stable cell attachment than the flat PDMS indicating that topography may overcome potential negative material influences.

During the 5‐day differentiation, areas appeared on the topography gradients (Figure 2d) and to a lesser extent on flat PDMS in an almost regular interspersed pattern comprising high densities of nuclei, that is, clustered cells. At these locations, spontaneous twitching occurred. This suggests that fusion of myoblasts had initiated at these points.

Myoblasts aligned to the topography in all sections of the gradient during adhesion and proliferation (Figure 2d, PM) although these cells had a random distribution on the TCP and flat PDMS controls. The alignment maintained during 2 and 5 days of differentiation, whereas on the controls (TCP and flat PDMS), myotubes appeared with a curved and disorganized morphology (one‐way ANOVA *p* < .0001 for PM, 2 and 5‐days DM). The alignment did not depend on the size of the topography as it occurred similarly at all wavelengths (Figure 3b). As expected, absence of topography, that is, flat surfaces caused a random orientation of myotubes of 43° ± 22° on TCP and 48° ± 21° on flat PDMS (Figure 3b, TCP and PDMS). The angle of cell orientation on all topographies improved with increasing fiber maturity. Prior to differentiation (PM), myoblasts had an average alignment of 9° in Section 1 (higher alignment angle of the sections). The other sections presented greater alignment, which decreased to an average of 4° after 5 days of differentiation.

### 3.2 Cells proliferate independent of topographical features

**Figure 4:**
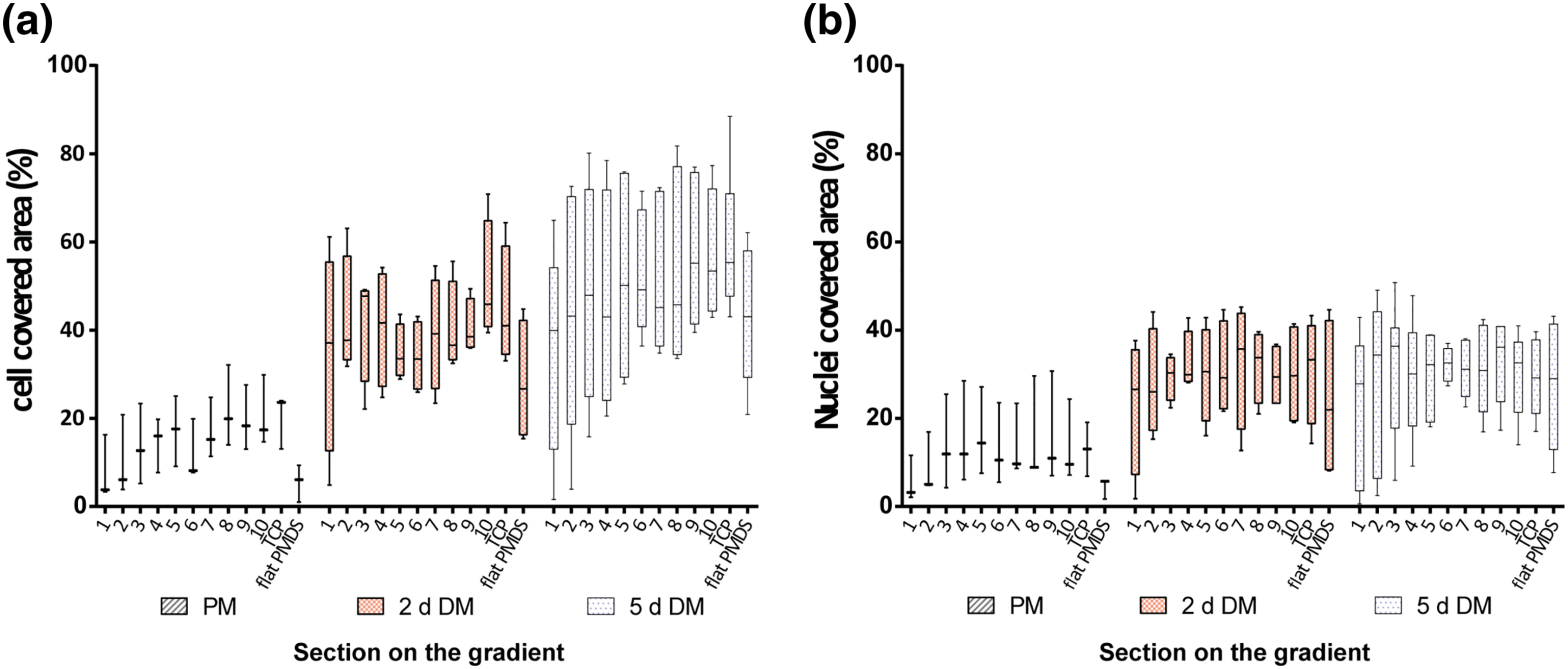
Graphical representation of data, percentage of area covered by cells expressing desmin (a) and DAPI (b) on the gradient, by box and whiskers plotting the minimum (smallest value) to the maximum (largest value) values and the line at the median. (a,b) Two‐way analysis of variance, time points after 3 days in proliferation medium, and 2 and 5 days in differentiation medium (PM, 2‐days DM and 5‐days DM) showed significant difference (*p* < .0001); no effect was presented between sections for area expressing desmin and DAPI. Data from three independent experiments and sample size duplicates (except PM with one sample per independent experiment). DM, differentiation medium; PM, proliferation medium [Colour figure can be viewed at http://wileyonlinelibrary.com]

The time between the seeding, at a relatively low density (5,000 per cm^2^), and the start of the differentiation process was 3 days. Visual inspection and fluorescent imaging showed that myoblasts had adhered to all topography features and appeared at higher density on larger topographies. Distribution of cells (Figure 4a) and nuclei (Figure 4b) was homogeneous along the length of each topography. However, quantitative determination of cell coverage in each section showed no differences, which was primarily due to the naturally occurring large variation between the independent experiments and triplicates. A similar tendency was observed for differentiating myoblasts, both after 2 and 5 days, for the coverage of the topographies with myotubes. Their coverage was homogeneous in all section albeit with a large variation, which concurred with the visual aspect of the cell coverage. After 5 days of differentiation, the fusion process had increased extensively, and as a result, gaps appeared between the myotubes due to acquiring a rounded shape. Therefore, the fraction area covered with cells did not reach 100%. Control TCP had a similar coverage as topography substrates, whereas flat PDMS had a low coverage of undifferentiated myoblasts, which remained lower than on the topographies during differentiation. This indicates that topographies, irrespective of size, augment proliferation and differentiation of myoblasts. It should be noted, however, that at 5‐days differentiation on flat PDMS, the myotubes tended to contract strongly and detach as sheets of cells from the substrate. This artificially reduced the measured coverage fraction. Undifferentiated cells at Section 1, smaller topography on the gradient, displayed a low cell coverage of 7.8 ± 7.3% similar as for flat PDMS on which the average covered area was 5.5 ± 4.2 %. In contrast, at time point of 5‐days DM, cells presented a very comparable percentage of cell area covered in Sections 2–10 varying from 42% to 60% whereas Section 1 only had a coverage of 35% (two‐way ANOVA *p* < .0001 between undifferentiated cells and five‐day‐old differentiatiated myotubes [5‐days DM]).

### 3.3 Myotube fusion and diameter does not depend on topography dimensions

**Figure 5:**
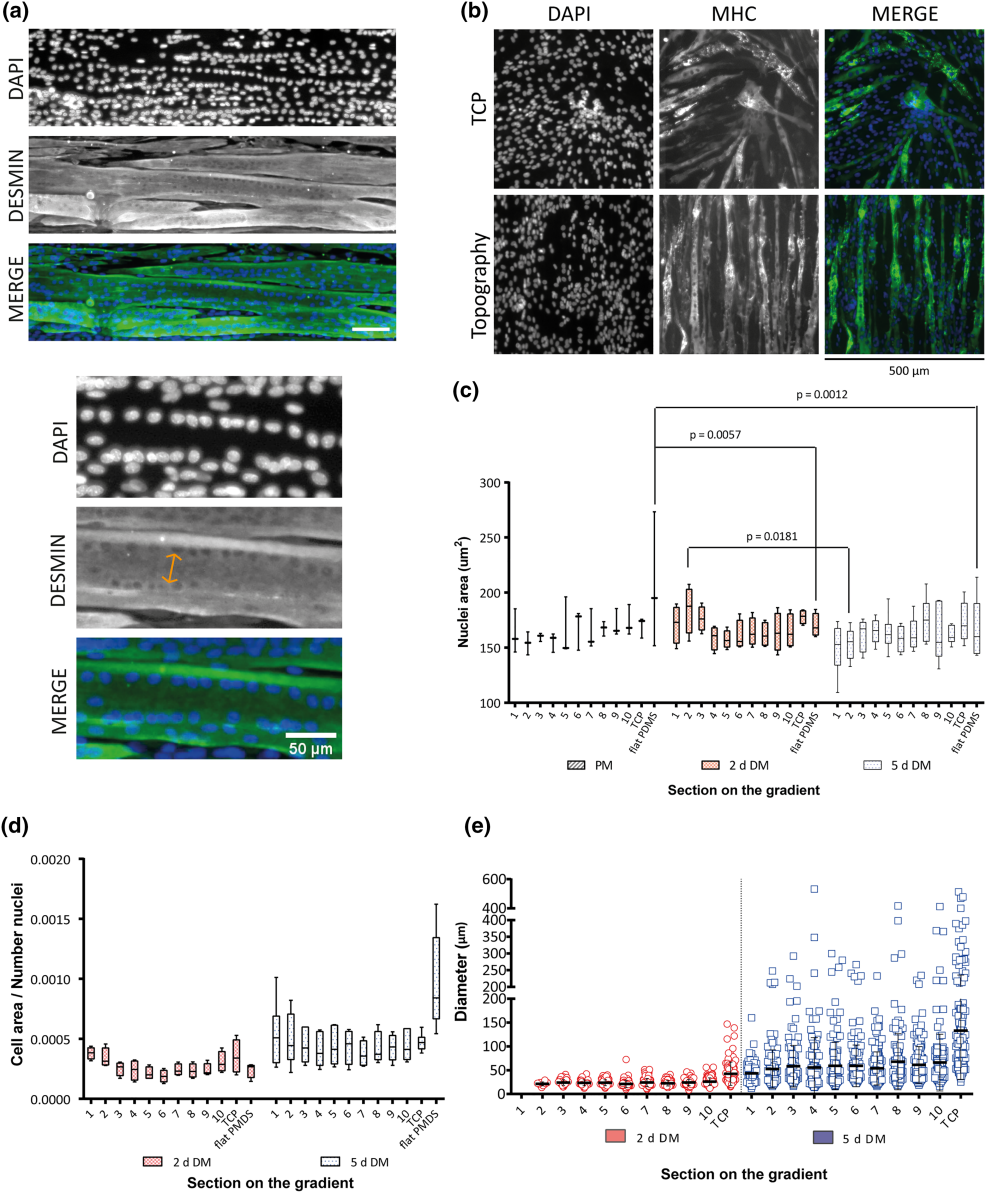
(a) Myotubes after 5 days of differentiation. Nuclei are aligned at the periphery of the myotube. Top to bottom: DAPI, desmin, and Merge picture. Scale bar 150 μm. Bottom: Zoom of image. Double‐arrow is pointing the aligned nuclei close to the myotube membrane. Sacale bar 50 μm. (b) Myosine heavy chain (MHC) staining after 3 days of differentiation showing early stages of maturation on the directional topography. Top TCP, bottom directional topography. (c) Nuclear area in the different sections while cells were undifferentiated (PM) and two and five differentiated (respectively, 2‐days DM and 5‐days DM). N.S. (d) Cell area covered by cells expressing desmin per number of nuclei in the particular section. Data are presented by box and whiskers plotting the minimum (smallest value) to the maximum (largest value) values and the line at the median. (Two‐way analysis of variance *p* = .0059) (e) Raw data was plotted to show the variation of the different cell diameters in the gradient. Wilcoxon matched‐pairs signed rank test *p* = .0020 between two differentiation time points. DM, differentiation medium; MHC, Myosine heavy chain; PM, proliferation medium; TCP, tissue culture polystyrene [Colour figure can be viewed at http://wileyonlinelibrary.com]

After 5 days of differentiation, formation and maturation of myotubes by fusion of myoblasts were visible as the appearance of multiple nuclei per cell (syncytia), which were located at the periphery of the myotubes. A zoomed in on the image showed how the nuclei are organizing in an aligned manner close to the myotube membrane where the sarcomere is developing (Figure 5a). Myotube maturity was observable and corroborated after 3 days of differentiation with myosin heavy chain staining (Figure 5b). The morphology and area of the individual nuclei were measured per every section of the directional gradient during proliferation and differentiation (Figure 5c). Flat controls showed a decreased nuclear area from time point PM to 2‐days DM (*p* = .0057) and from PM to 5‐days DM (*p* = .0012). Section 2 had also a decrease in nuclear area from 2‐days DM to 5‐days DM (*p* = .0181) (Tukey's multiple comparison test Figure 5c).

Myotube maturity was also determined by considering the growth of the sarcomere after fusion. Cell area of cells expressing desmin, an intermediate filament protein and part of the sarcomere, was used to calculate the ratio between cell areas over the number of nuclei found in the same area measured. This gave, as a result, the fusion ratio from 2 to 5 days of differentiating myotubes (Figure 5d). The ratio of cell area per nuclei number showed an increased over differentiation time (two‐way ANOVA *p* = .0059). The flat control in comparison with all sections had a significant increase suggesting a highest fusion ratio (Sidak's multiple comparisons test *p* < .0001). This corresponds with the results observed at 2 days of differentiatiating myotubes, where myotubes were not present at the flat PDMS, but after 5 days of differentiating, myotubes were clearly visible on that substrate (Figure 2d).

Flat PDMS and Section 1 at 2 days of differentiation showed low myotube formation and low capacity to maintain attached to the substrates over time. For this reason, this topography was excluded from the diameter evaluations (Figure 5e). Although, after 2 days of differentiation, myotubes had started to form; their diameters were relatively small, that is, below 50 μm (Figure 5e). After 5 days of differentiation, myotube maturation had proceeded as assessed by the increase in diameter, whereas on TCP, diameters were still larger, reaching up to 513 μm with an average of 133 ± 102 μm (Kruskal–Wallis test *p* = .0350). The fusion and maturation process did not appear to have reached maximal levels because the variation of diameter of individual tubes was considerable ranging from ~10 to ~100 μm for the smallest topography section, ~10 to 400 μm for the largest topography section, and ~10 to ~500 μm on TCP controls. Myotubes in topography Section 10, had a maximum diameter of 412 μm with an average of 66 ± 59 μm. However, the diameter had a significant increase in size from 2 to 5 days of differentiating myotubes (Wilcoxon matched‐pairs signed rank test *p* = .002).

## 4 DISCUSSION

In this study, we showed that directional topography gradients induce alignment of human myoblasts and myotubes to the length of the topographies. The aligned myotubes contracted spontaneously irrespective of the topography section. Myoblasts and myotube alignment has been achieved by different systems in 2D and 3D (Choi et al., 2008; Duffy et al., 2016; Juhas, Engelmayr, Fontanella, Palmer, & Bursac, 2014; Koning, 2012; Nagase et al., 2017; Sengupta et al., 2012; Takahashi et al., 2015; Takahashi, Shimizu, Nakayama, Yamato, & Okano, 2013), but an evaluation of different features within a same substrate, that is, in a gradient surface has not been reported to date. In addition, well‐known systems with protein lines and spacing (Duffy et al., 2016; Sengupta et al., 2012) and bio‐artificial muscle constructs (Madden et al., 2015) have not determined the exact relation of topography and human skeletal muscle cell behavior. The 2D system used in this study added value to the understanding of the influence of topography driving the alignment and differentiation of human myoblasts.

Our directional topography gradient showed that human myoblasts aligned in all dimensions of topography (i.e., nanometer to micrometer) and did not depend on wavelength or amplitude. Other pattern systems (Duffy et al., 2016) made by ECM proteins such as laminin, collagen, and fibronectin showed human cells aligning in patterns ranging from 10 to 20‐μm line spacing and line widths 50 to 200 μm. Our system had even smaller features, 1.5 μm in wavelength, which suggests that human cells respond to an extensive range of topographies that result in alignment.

The influence of topography on the cell orientation and alignment also influenced the fusion and maturation of the myotubes over time. After 2 days of differentiation in Section 1, (λ = 1,520 nm and A = 176 nm) no myotubes were formed. Instead, after 2 days of differentiation, a mixed population of cells (myoblasts and myotubes) along the gradient was found. After 5 days of differentiation, tube formation was extended all along the gradient independently of section, that is, myotubes had matured. Myotube formation was first observed in sections with larger size features but that did not inhibit the cell migration and later myotube formation in sections of smaller topography features. Once the cell layer on the gradient is confluent, myotube formation becomes a regular fusion process irrespective of the topography features. Cells proliferated and differentiated in an aligned manner and were not restricted or confined to the wavelength size once matured.

In contrast, flat PMDS inhibited but did not prevent attachment of myoblasts and myotube formation. After 2 days of differentiation, flat PDMS showed lower cell population. Cell proliferation and differentiation were reduced, and cell detachment occurred faster. However, after 5 days of differentiation, there was an increase of myotube formation, which suggests that a satellite cell fraction continues to proliferate during the fusion process to myotubes. Similarly, cells did not attach strongly to section 1 (λ = 1,520 nm and A = 176 nm) implying that this topography is sensed by cells as flat PDMS. Therefore, this section of the gradient seems to be the topographical limit that human myoblasts can sense properly. A different alignment limit has been reported for C2C12 cells where smaller features (λ = 830 nm and [A] = 100 nm) are less efficient in alignment (Grigola et al., 2014). In both cases, alignment improved with increase of confluency. However, these results confirm the different cell response to alignment between murine origin and human myoblasts.

In vitro cell maturation has been evaluated by observing the nuclei pushed to the sides and close to the sarcolemma after 21 days in culture in a bioartificial muscle system (Koning, 2012). In our directional topography gradient, topographies are larger than those in Section 1 augmented cell attachment, proliferation, and differentiation because the nuclei were pushed to the sides and were close to the sarcolemma after 5 days. In addition, spontaneous contraction was observed already after 4 days of differentiation presenting a high maturity state of the cells in vitro and rarely reported in a 2D system (Guo et al., 2014; Video S1).

Spontaneous contractions of fused myoblasts in vitro was reported as early as 1960 (Capers, 1960) for chicken and for human myoblasts in 1975 (Askanas & Engel, 1975). Tanaka et al. showed that calcium signaling via dihydropyridine receptors, ryanodine receptors, and triadin regulate spontaneous and innervated contractions (Tanaka, Furuya, Kameda, Kobayashi, & Mizusawa, 2000). Most likely, calcium transients are also responsible for the myotubes' spontaneous contractions that we observed in the directional topography gradients.

This spontaneous contraction might underlie the detachment of the cells from the substrates. Cell delamination is a common challenge that is acknowledged by several groups working on 2D systems (Duffy et al., 2016; Murray et al., 2016; Vandenburgh et al., 2008). Lack of surface coating renders cell attachment challenging because a coating with (ECM) proteins might improve cell adhesion and differentiation (Ngandu Mpoyi et al., 2016). Our topography was devoid of a coating but showed different behavior once compared with flat PDMS suggesting a positive influence on the cell adhesion by topography. Future investigations focus on the deposition of basement membrane components by myoblasts on the topographies. Cells started to delaminate from the smaller topographies section after 5 days differentiation, but larger topography features restrained attached myotubes. On the other hand, flat PDMS started to show monolayer detachment from the whole surface after 5 days of differentiation. A monolayer of cells cultured on a PDMS substrate attached strongly between each other (Murray et al., 2016) suggesting that delamination of cells was produced by the collapse of some myotubes, producing a sheet pealing‐off effect more strongly present on surfaces without our topography.

In addition, our approach considered the topographical architecture of the skeletal muscle but did not consider physiological muscle stiffness. The stiffness of the PDMS (90 MPa) was higher than muscle (12 KPa; Schaap‐Oziemlak, Kühn, van Kooten, & van Rijn, 2014; Zhou et al., 2018), which affects cell attachment and behavior. Stiffer substrates increase the proliferation of myogenic progenitor cells (Engler et al., 2004). Our topographical and flat PDMS surfaces have the same stiffness suggesting that indeed topography is influencing cell attachment and orientation whereas the stiffer TCP is promoting proliferation.

Topographical systems in 2D resemble diameter sizes of myotubes created in 3D systems through the generation of myotubes of approximately 20 μm in diameter after 1 and 4 weeks of differentiation (Choi et al., 2008; Madden et al., 2015; Takahashi et al., 2013), which do not resemble the natural diameter size of an adult human of 100 μm (Alberts et al., 2002). Our linear topographical gradient generated myotubes with an average diameter of 66 ± 59 μm (Section 10), which is close to the desired physiological value and larger than reported before to the best of our knowledge. However, there is a discrepancy in the literature whether the diameters of the myotubes are larger in the aligned topography or on the flat surfaces (Choi et al., 2008; Takahashi et al., 2013). In our case, myotubes produced by the topography had a significant smaller diameter than the control TCP. There is, however, a large spread in the diameters considered for the analysis because the myotubes, once matured, were branching and interconnecting, forming larger myotubes as large as 412 μm. Murray et al. showed that differentiation of C2C12 myotubes behaved differently due to the material substrate; PDMS showed slower differentiation in comparison with TCP (Murray et al., 2016). In addition, the discrepancy between the production of myotubes on topography and flat surfaces might be related with the techniques used to produce the myotubes (Choi et al., 2008; Takahashi et al., 2013).

## 5 CONCLUSIONS

Four key findings arise from this research. Firstly, human myoblasts aligned and differentiated irrespective of topography size (range λ = 1,520 nm and A = 176 nm to λ = 9,934 nm and A = 2,168 nm). In addition, the differentiation process was only inhibited or delayed by nanosized topography or flat surfaces. Furthermore, aligned myotubes were able to contract spontaneously, and nuclei were organized on the sarcomere periphery. Finally, myotubes generated in our system had an average diameter of 66 ± 59 μm, which corresponds to the physiological muscle myotubes.

This directional topographical gradient helped to understand the topography associated with that of the natural skeletal muscle. Entangling the natural geometry that resides in the human skeletal muscle ECM in combination with the design of biodegradable materials will help the construction of scaffolds resembling the biophysical and biochemical properties of the natural tissue. Therefore, further understanding of how topography influences protein adhesion and cellular processes of myoblasts is still required.

## CONFLICT OF INTEREST

The authors declare the following competing financial interest(s): P. v.R also is co‐founder, scientific advisor, and shareholder of BiomACS BV, a biomedical‐oriented screening company.

## Supporting information


**Figure S1:** After two days in differentiation medium (2 d DM) a mixed cell population of myoblasts and myotubes emerged on the directional topography. In the left microcraph, a zoomed in from the middle picture, it is visible the myoblast population and in the right microcraph, it is a zoomed in of the aligned myotubes. Desmin (green) and DAPI (blue).

**Video S1:** Aligned myotubes are spontaneously contracting. Both videos are taken after four days of differentiation. Videos were taken with an inverted microscope Leica DM IL LED equipped with DFC 425C CCD camera. Software LAS V4.5 (Leica Microsystems CMS GmbH, Wetzlar, Germany). Videos were eddited with Adobe Premiere Pro.
