# Supplementary figures and images for: Directional topography gradients drive optimum alignment and differentiation of human myoblasts
Source: J Tissue Eng Regen Med. 2019 Nov 10;13(12):2234–45. doi: 10.1002/term.2976 (PMC6973069; doi:10.1002/term.2976)

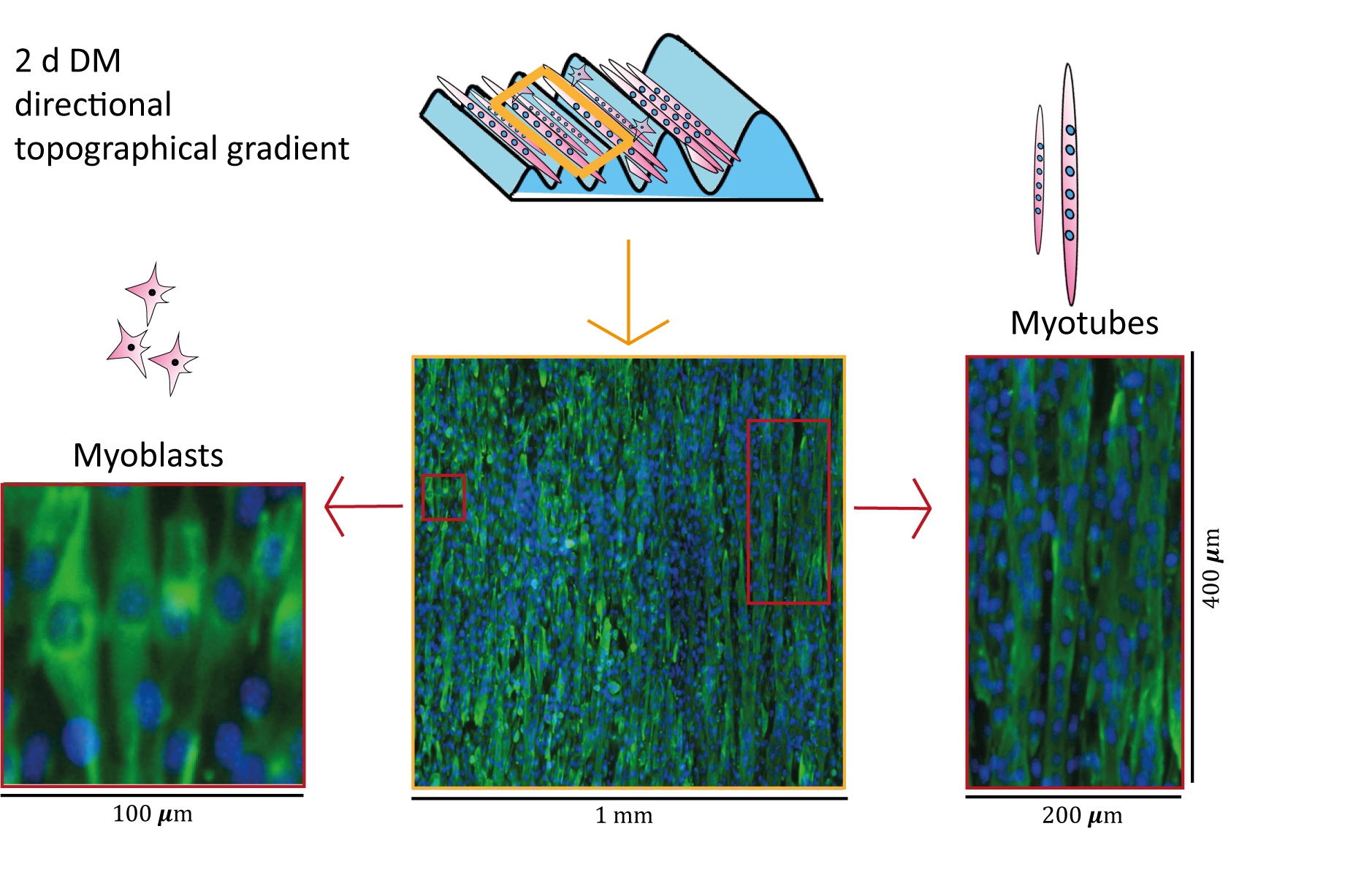

Supplement: Supplementary file 1 — Figure S1: After two days in differentiation medium (2 d DM) a mixed cell population of myoblasts and myotubes emerged on the directional topography. In the left microcraph, a zoomed in from the middle picture, it is visible the myoblast population and in the right microcraph, it is a zoomed in of the aligned myotubes. Desmin (green) and DAPI (blue). [file TERM-13-2234-s001.tif]
